# Postprandial attenuation of gastric slow waves in anxiety–depression, with and without functional dyspepsia: associations with heart rate variability and sleep quality

**DOI:** 10.3389/fmed.2026.1890716

**Published:** 2026-07-27

**Authors:** Lin Du, Jie Yang, Li Jiang, Guiying Zeng, Yanping Shu, Bin Bi

**Affiliations:** 1 Department of Gastroenterology, The Affiliated Hospital of Guizhou Medical University, Guiyang, Guizhou, China; 2 Department of Gastroenterology, Anshun People's Hospital, Anshun, Guizhou, China; 3 Department of Gastroenterology, The Second People's Hospital of Guizhou Province, Guiyang, Guizhou, China; 4 Department of Gastroenterology, Qixingguan District People's Hospital of Bijie City, Bijie, Guizhou, China; 5 Department of Clinical Psychology, The Second People's Hospital of Guizhou Province, Guiyang, Guizhou, China

**Keywords:** anxiety, depression, electrogastrography, functional dyspepsia, gastric slow waves, heart rate variability, postprandial response index, sleep quality

## Abstract

**Background and aims:**

Functional dyspepsia (FD) commonly coexists with anxiety, depression, and sleep disturbances. However, it remains unclear whether the burden of psychological symptoms and sleep disturbances is associated with autonomic regulation and meal-induced gastric myoelectrical responses, particularly in individuals without overt FD. This study aimed to explore the relationships among heart rate variability (HRV), sleep quality, and postprandial electrogastrographic changes in adults with symptoms of anxiety–depression (AnxDep), with or without FD.

**Methods:**

A single-center cross-sectional observational study enrolled 253 participants across four clinically defined phenotypes: healthy controls (*n* = 75), FD-only (*n* = 45), mild-to-moderate anxiety–depression without FD (*n* = 60), and severe anxiety–depression with FD (*n* = 73). The design was not a complete factorial design; therefore, group comparisons were interpreted as descriptive phenotype comparisons rather than as estimates of the interaction between anxiety–depression severity and FD status. Anxiety, depression, sleep quality, and FD symptoms were assessed using the self-rating anxiety scale (SAS), self-rating depression scale (SDS), Pittsburgh Sleep Quality Index (PSQI), and a structured FD scale. Surface electrogastrography (EGG) was recorded before and after a standardized meal, and 24-h HRV was monitored. Postprandial response index (PRI) was calculated as the postprandial-to-preprandial normogastria ratio and was analyzed as an exploratory research index. Statistical analyses included group comparisons, correlations, collinearity-aware regression models, and sensitivity analyses; causal mediation analysis was not performed.

**Results:**

HRV indices significantly differed across groups (all *p* < 0.001). Lower RMSSD and HF values were observed in the FD and anxiety–depression groups, with the lowest vagal-related indices in the severe anxiety–depression with FD group. LF/HF was reported only as a relative spectral ratio and was not interpreted as a direct measure of sympathovagal balance. The anxiety–depression without FD group had preserved absolute normogastria values but a reduced PRI (0.874 ± 0.066 vs. 1.145 ± 0.086 in controls), with all participants in this group showing PRI < 1. In sensitivity analyses, the difference in PRI between the anxiety–depression without FD group and controls persisted after adjustment for BMI or weight, medication exposure, and recruitment source, and after excluding participants using SSRI/SNRIs or sleep medications.

**Conclusion:**

Anxiety–depression symptoms were associated with reduced vagal-related HRV, poorer sleep quality, and a blunted postprandial gastric slow-wave response. In the anxiety–depression without FD group, a PRI < 1 reflected a blunted meal-related slow-wave response despite preserved absolute normogastria values. PRI should be considered an exploratory research endpoint requiring prospective validation.

## 1 Introduction

Functional dyspepsia (FD) is a common disorder of the gut–brain interaction (43, 44). Under Rome IV criteria, FD is defined by chronic or recurrent postprandial fullness, early satiation, epigastric pain, or epigastric burning without a structural, systemic, or metabolic explanation (1, 2). Because many FD symptoms are meal-related, the fasting-to-postprandial transition is a clinically relevant window during which gastric accommodation, antral motor activity, slow-wave rhythm, and sensory feedback must be coordinated (3–9).

Surface electrogastrography (EGG) records gastric myoelectrical rhythm from the body surface. Normal gastric slow-wave activity occurs at approximately three cycles per minute and supports the timing of gastric contractions, although surface EGG is not a direct measure of gastric emptying or contractile force (10, 11). FD has been associated with reduced normogastria and increased bradygastria or tachygastria, but many previous studies have focused on patients who already meet FD criteria (12–17).

Psychological symptoms and poor sleep are frequent in FD and may interact with gastric physiology through brain–gut and autonomic pathways (18–35, 45, 46). RMSSD and high-frequency (HF) HRV are commonly used as vagal-related indices, whereas LF/HF should be treated as a relative spectral ratio rather than a direct measure of sympathovagal balance (36–39). Sleep disturbance may further affect autonomic tone, arousal, pain processing, inflammatory signaling, and interoceptive regulation.

The key knowledge gap is that psychological symptoms, sleep quality, HRV, and EGG have rarely been assessed in the same participants, particularly in individuals with anxiety–depression symptoms but no overt FD. Static preprandial or postprandial EGG percentages may overlook a dynamic abnormality in the meal response. We therefore defined PRI as the ratio of postprandial to preprandial normogastria percentage to describe whether normal slow-wave proportion increased or decreased after meal stimulation.

This study evaluated anxiety, depressive symptoms, sleep quality, HRV, and EGG in healthy controls, FD-only patients, individuals with mild-to-moderate anxiety–depression without FD, and individuals with severe anxiety–depression with FD. We aimed to describe phenotype-level differences and examine associations among psychological symptoms, sleep quality, vagal-related HRV, and meal-induced gastric slow-wave responses. Because of the cross-sectional design, neither temporal order nore causality could be inferred.

## 2 Methods

### 2.1 Study design, participants, and grouping

This single-center cross-sectional observational study enrolled 178 patients from gastroenterology and psychological medicine outpatient clinics and inpatient units, together with 75 healthy volunteers, between March 2024 and November 2025. This study was reported in accordance with the STROBE statement (47). Participants were assigned to four clinically defined phenotypes according to prespecified anxiety–depression symptom severity and Rome IV FD status: healthy controls (*n* = 75), FD without clinically significant anxiety–depression (*n* = 45), mild-to-moderate anxiety–depression without FD (*n* = 60), and severe anxiety–depression with FD (*n* = 73). Anxiety–depression severity was classified using the prespecified Zung standard-score categories in the clinical protocol: no clinically significant anxiety–depression was defined as SAS < 50 and SDS < 53; mild-to-moderate anxiety–depression symptoms were defined by the highest SAS or SDS category falling in the mild or moderate range (SAS 50–69 or SDS 53–72); and severe anxiety–depression symptoms were defined by a severe-range SAS or SDS or SDS score (SAS ≥ 70 or SDS ≥ 73). The higher severity category indicated by either the SAS or SDS was used for phenotype assignment.

The study was not designed as a complete 2×2 factorial study because mild-to-moderate anxiety–depression with FD and severe anxiety– depression without FD groups were not available in sufficient numbers under the clinical recruitment protocol. These phenotypes were selected to reflect the main clinical presentations encountered in the participating departments—FD without clinically significant anxiety–depression, anxiety–depression without overt FD, and severe anxiety–depression with comorbid FD—rather than to test a full factorial interaction model. Therefore, analyses were interpreted as descriptive comparisons among clinically defined phenotypes, and no interaction between anxiety–depression severity and FD status was estimated. Recruitment source was recorded as healthy volunteer, outpatient, or inpatient. Source differences were treated as potential selection factors and were considered in sensitivity analyses and limitations.

### 2.2 Sample size and design considerations

This exploratory observational study enrolled all eligible participants during the prespecified recruitment period; therefore, no formal *a priori* sample-size calculation was performed. We did not calculate retrospective power from the observed effect estimates. Instead, an achieved-sample sensitivity analysis was performed with G*Power 3.1.9.7 to determine the smallest standardized effects detectable at *α* = 0.05 and 80% power (two-sided for the pairwise test) (40). With a total sample size of *N* = 253 in a four-group fixed-effects omnibus test, the minimum detectable effect size was Cohen’s *f* = 0.209. For the key comparison between healthy control and individuals with mild-to-moderate anxiety–depression without FD (*n* = 75 and *n* = 60), the minimum detectable effect size was Cohen’s d = 0.489 at a nominal *α* = 0.05 (d = 0.611 with a Bonferroni α = 0.0083 for six pairwise contrasts). For fixed-model linear regression testing one focal psychological/sleep predictor added to age, sex, and BMI (one tested predictor; four total predictors), the minimum detectable incremental effect was Cohen’s f^2^ = 0.031 in the full sample and f^2^ = 0.068 in the FD subgroup (*n* = 118).

These sensitivity calculations indicate that the achieved sample had sensitivity to detect small-to-moderate omnibus and regression effects and moderate pairwise effects, whereas very small effects and sparse medication-class or subgroup contrasts may have been missed. To reduce overfitting in the relatively small FD-only group and avoid unstable coefficients in highly collinear models, the main regression strategy emphasized parsimonious models. SAS, SDS, and PSQI were modeled separately, each adjusted for age, sex, and BMI. The FD symptom models included 118 participants and four predictors (29.5 observations per predictor). Full models, including highly collinear psychological and sleep predictors, were retained only as exploratory supplementary analyses.

### 2.3 Eligibility criteria, medication assessment, and ethics approval

Eligible participants were 18–65 years old and able to complete psychometric questionnaires and physiological testing. The age range was selected to include adult participants while reducing confounding from pediatric physiology and older-age comorbidities or age-related autonomic changes. Exclusion criteria were organic gastrointestinal disease confirmed by endoscopy, imaging, or laboratory testing; severe cardiac, cerebral, pulmonary, renal, or endocrine disease; pregnancy or lactation; and recent use of drugs likely to affect gastric motility or autonomic function unless discontinuation before testing was documented as clinically sufficient. Participants with recent use of prokinetic agents, anticholinergic drugs, or tricyclic antidepressants were excluded unless discontinuation before testing was documented as at least 72 h or at least five half-lives, where clinically applicable; current use of these drug classes was absent in the analytic sample.

Medication exposure was abstracted from available source records, including clinical interview records and medication records, when present, and rechecked at the class level during data verification. Available medication classes included SSRI, SNRI, benzodiazepines, non-benzodiazepine hypnotics, antipsychotics, mood stabilizers, prokinetics, anticholinergic drugs, tricyclic antidepressants, proton-pump inhibitors, and H2 blockers. Medication categories were not mutually exclusive. Dose, duration, adherence, and exact timing of last intake were not consistently available; therefore, medication-related analyses were treated as sensitivity analyses rather than complete control of medication confounding.

Two experienced clinicians conducted structured interviews to confirm eligibility and group allocation. Severe, unstable psychiatric illness was excluded. Prior psychiatric history and major recent stressors within the preceding 6 months were recorded descriptively during clinical interview; these variables were not used for causal adjustment because standardized timing, severity, and life-event scale data were not available. The study protocol was approved by the institutional ethics committee (approval number SEY-LL-BG-202311-27-1.0), and all participants provided written informed consent.

### 2.4 Clinical and questionnaire assessments

Demographic variables included age, sex, height, weight, and body mass index (BMI). Anxiety was measured with the self-rating anxiety scale (SAS), and depressive symptoms with the self-rating depression scale (SDS) (41, 42). Both instruments contain 20 self-rated items scored on a four-point frequency scale, with higher scores indicating more severe symptoms. Sleep quality was measured using the Pittsburgh Sleep Quality Index (PSQI), which covers subjective sleep quality, sleep latency, sleep duration, sleep efficiency, sleep disturbance, sleep medication use, and daytime dysfunction35-37. Higher PSQI scores indicate poorer sleep quality.

### 2.5 Dyspeptic symptom assessment and FD diagnosis

Dyspeptic symptoms were evaluated with a structured FD symptom score covering upper abdominal pain, upper abdominal distension, postprandial fullness, early satiation, belching, nausea, retching, vomiting, appetite loss, bloating, and visible abdominal distension. For each symptom, severity and frequency were rated by the participant and verified by the clinician. Higher total scores indicated greater dyspeptic symptom load. FD was diagnosed according to Rome IV gastroduodenal criteria after structured clinical assessment and exclusion of relevant organic disease (1, 2).

### 2.6 Standardized test meal, electrogastrography, and postprandial response indices

Participants underwent EGG testing after an overnight fast of at least 8 h. Caffeine and alcohol within 24 h, smoking on the morning of testing, and strenuous activity within 24 h were restricted and checked verbally before testing. All participants were provided the same standardized test meal used in the laboratory protocol, consisting of steamed bread (100 g), a boiled egg (50 g), and milk (250 mL), with an estimated total energy content of approximately 430 kcal, including approximately 58 g of carbohydrate, 19 g of protein, and 14 g of fat. Participants were instructed to consume the meal within 15 min; meal provision and pre-test restrictions were treated as protocol requirements rather than formal adherence outcomes.

Gastric myoelectrical activity was recorded by surface EGG using a Meda electrogastrography system with standard epigastric three-electrode placement. After a 30-min fasting baseline recording, participants consumed the standardized meal and then underwent a 30-min postprandial recording. Signals were summarized using the laboratory processing pipeline. The analysis band was 0.5–9.0 cycles per min (cpm), with bradygastria defined as 1.0–2.0 cpm, normogastria as 2.0–4.0 cpm, and tachygastria as 4.0–9.0 cpm. Segments with motion artifact, electrode drift, or excessive noise were removed before spectral summary. A structured laboratory quality-control review was performed, and borderline or artifact-prone recordings were checked by a second reviewer; disagreements about signal acceptability or artifact removal were resolved by consensus. Since this consensus review was not designed as a formal inter-rater reliability study, no inter-rater reliability coefficient is reported.

The extracted indices were the proportions of normogastria (N%), bradygastria (B%), and tachygastria (T%) before and after the meal. PRI was calculated as postprandial N% divided by preprandial N%. Bradygastria and tachygastria ratios were calculated similarly. Surface EGG was considered a measure of gastric rhythm, not a direct measure of gastric emptying, accommodation, or contractile force (10–12, 17). PRI was analyzed as an exploratory meal-response index rather than an established diagnostic threshold.

### 2.7 Heart rate variability assessment

Autonomic function was assessed by 24-h electrocardiographic monitoring using an oranGer ambulatory ECG monitor (Meda electrogastrography system, Zhejiang Meda Perth Medical Technology Co., Ltd., Hangzhou, China; oranGer ambulatory ECG monitor, Oranger, Tianjin, China). After skin preparation with 75% alcohol to reduce electrode interference, ambulatory ECG monitoring was initiated between 8:00 and 8:30 a.m. and continued for 24 h. Participants were instructed to avoid strenuous activity during the 24 h before monitoring and to follow testing instructions during recording. HRV variables included SDNN, RMSSD, HF power, LF power, and LF/HF. RMSSD and HF were selected as the main vagal-related indices. LF/HF was analyzed only as a relative spectral ratio and not as a pure measure of sympathovagal balance (36–39).

### 2.8 Statistical analysis

Continuous variables are presented as mean ± standard deviation or median and interquartile range where appropriate, and categorical variables as frequencies and percentages. Between-group comparisons used the Kruskal-Wallis rank-sum test, with Dunn-Bonferroni correction for pairwise comparisons where relevant. Categorical variables were compared using Pearson chi-square tests. Preprandial–postprandial changes in EGG indices were assessed using paired Wilcoxon tests.

Spearman correlation analysis examined relations among psychological scores (SAS and SDS), sleep quality (PSQI), HRV variables (SDNN, RMSSD, HF, LF, and LF/HF), and EGG ratio variables (PRI, bradygastria ratio, and tachygastria ratio). Since SAS, SDS, and PSQI were strongly interrelated, variance inflation factors (VIFs) were calculated, and separate parsimonious ordinary least-squares models were prioritized. PRI, RMSSD, and FD symptom scores were treated as continuous outcomes; each main model included one focal SAS, SDS, or PSQI predictor plus age, sex, and BMI. In Supplementary Table S8, the group was entered as three-indicator variables with healthy controls as the reference category: FD-only, mild-to-moderate anxiety–depression without FD, and severe anxiety–depression with FD. The reported group contrasts are unstandardized indicator coefficients. Age, BMI, and weight were continuous; sex and medication-use variables were entered as binary indicators. The exact covariate set and analysis sample for each sensitivity model are specified in Supplementary Table S8. Sensitivity analyses additionally adjusted for weight or medication exposure and repeated key comparisons after excluding SSRI/SNRI users, sleep-medication users, or inpatient participants.

No mediation analysis was included in the main analysis because temporal ordering cannot be verified in cross-sectional data. All regression, correlation, and sensitivity results were interpreted as associations rather than causal pathways.

## 3 Results

### 3.1 Participant characteristics and clinical symptom profiles

The study included 253 participants: 75 healthy controls, 45 patients with FD but without clinically significant anxiety–depression, 60 patients with mild-to-moderate anxiety–depression without FD, and 73 patients with severe anxiety–depression and FD. Sex distribution, age, and height were similar across groups. Weight and BMI differed across groups, with lower values in the anxiety–depression groups, especially in the severe anxiety–depression with FD group. This difference was considered physiologically relevant and was addressed through covariate adjustment and sensitivity analyses.

SAS and SDS scores were low in controls and in the FD-only group, increased in the mild-to-moderate anxiety–depression without FD group, and were highest in the severe anxiety–depression with FD group. FD symptom scores were zero in controls and in the anxiety–depression without FD group, elevated in the FD-only group, and highest in the severe anxiety–depression with FD group. PSQI scores showed a similar clinical gradient, and sleep-medication use was more frequent in the anxiety–depression groups (Table 1).

**Table 1:** Demographic characteristics, clinical scale scores, sleep quality, and recruitment information across groups.

Variable	Healthy controls *N* = 75	FD-only *N* = 45	Mild-to-moderate AnxDep without FD *N* = 60	Severe AnxDep with FD *N* = 73	*p* value
Male sex, n (%)	30 (40%)	19 (42%)	26 (43%)	34 (47%)	0.900
Age, years	47.04 ± 12.15	45.16 ± 10.42	46.32 ± 12.48	46.86 ± 12.05	0.600
Height, cm	165.40 ± 7.54	164.60 ± 7.10	164.80 ± 7.46	165.41 ± 7.92	>0.900
Weight, kg	60.29 ± 7.82	61.91 ± 6.75	58.27 ± 7.90	55.86 ± 6.51	<0.001
BMI, kg/m^2^	21.74 ± 2.17	23.12 ± 1.69	21.03 ± 2.13	20.12 ± 1.71	<0.001
SDS score	36.28 ± 6.12	36.16 ± 6.10	59.30 ± 4.55	81.27 ± 5.51	<0.001
SAS score	36.65 ± 6.19	35.06 ± 5.79	59.46 ± 5.81	79.98 ± 5.58	<0.001
PSQI total score	4.15 ± 1.61	7.51 ± 2.23	12.20 ± 2.66	16.07 ± 2.67	<0.001
FD symptom score	0.00 ± 0.00	34.47 ± 3.02	0.00 ± 0.00	53.79 ± 8.97	<0.001
Any psychotropic medication, n (%)	1 (1%)	3 (7%)	42 (70%)	68 (93%)	<0.001
Sleep medication use, n (%)	1 (1%)	3 (7%)	29 (48%)	56 (77%)	<0.001
Inpatient recruitment, n (%)	0 (0%)	8 (18%)	23 (38%)	49 (67%)	<0.001

Values are mean ± SD or n (%). Between-group comparisons used Kruskal-Wallis rank-sum tests for continuous variables and Pearson chi-square tests for categorical variables. AnxDep, anxiety–depression.

### 3.2 Gastric myoelectrical rhythm profiles

**Figure 1:**
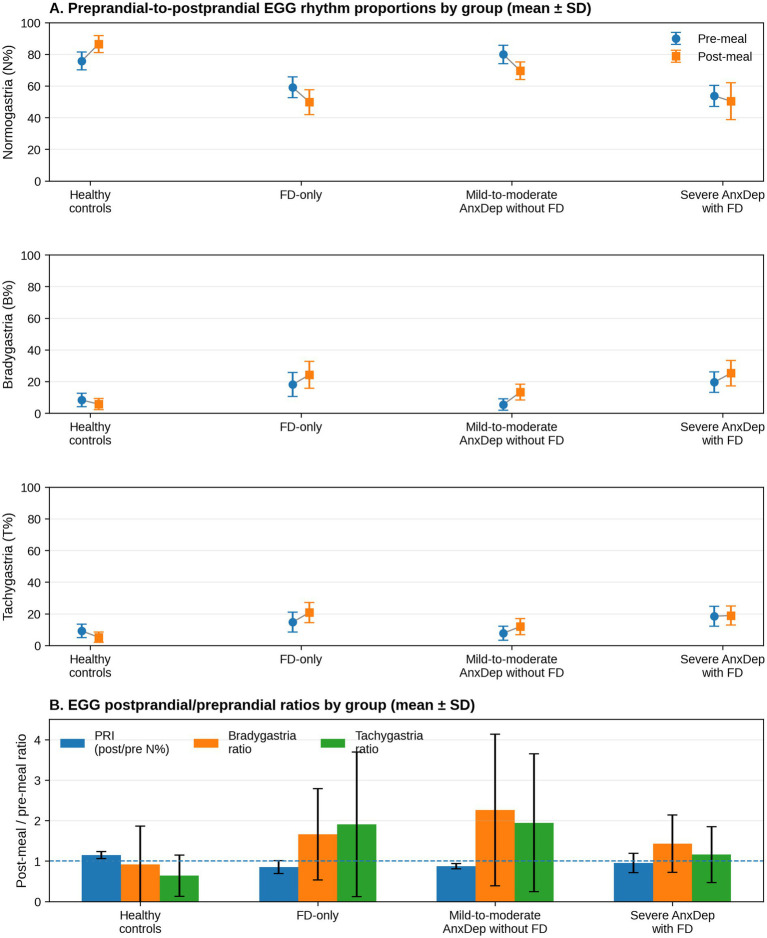
Summary of electrogastrography changes. Preprandial-to-postprandial changes in electrogastrographic rhythm parameters across groups. **(A)** Preprandial and postprandial proportions of normogastria (N%), bradygastria (B%), and tachygastria (T%) across the four clinical phenotypes. Values are presented as mean ± SD. **(B)** Postprandial/preprandial EGG ratio variables across the same four groups, including the postprandial response index (PRI; postprandial N% divided by preprandial N%), bradygastria ratio, and tachygastria ratio. Values are presented as mean ± SD. The dashed horizontal line indicates a ratio of 1, corresponding to no postprandial change.

EGG variables showed different fasting and postprandial profiles across groups (Table 2; Figure 1). In controls, meal stimulation increased normogastria and reduced dysrhythmic activity. The FD-only group and the severe anxiety–depression with FD group had lower preprandial normogastria and higher dysrhythmic proportions before meal stimulation, followed by further impairment or limited recovery after the meal.

**Table 2:** Electrogastrography rhythm parameters and postprandial/preprandial ratios.

Variable	Healthy controls *N* = 75	FD-only *N* = 45	Mild-to-moderate AnxDep without FD *N* = 60	Severe AnxDep with FD *N* = 73	*p* value
Preprandial normogastria (N%)	75.80 ± 5.65	59.16 ± 6.57	79.90 ± 5.80	53.76 ± 6.69	<0.001
Postprandial normogastria (N%)	86.48 ± 5.37	49.80 ± 7.86	69.64 ± 5.51	50.36 ± 11.70	<0.001
Preprandial bradygastria (B%)	8.32 ± 4.24	18.14 ± 7.56	5.48 ± 3.62	19.60 ± 6.52	<0.001
Postprandial bradygastria (B%)	5.74 ± 3.51	24.23 ± 8.55	13.29 ± 4.99	25.28 ± 8.09	<0.001
Preprandial tachygastria (T%)	9.20 ± 4.28	14.72 ± 6.29	7.77 ± 4.42	18.50 ± 6.25	<0.001
Postprandial tachygastria (T%)	5.18 ± 3.26	20.82 ± 6.38	11.92 ± 5.13	18.92 ± 5.93	<0.001
PRI (post/pre normogastria)	1.145 ± 0.086	0.851 ± 0.160	0.874 ± 0.066	0.950 ± 0.239	<0.001
Bradygastria ratio (post/pre)	0.914 ± 0.948	1.663 ± 1.129	2.260 ± 1.874	1.428 ± 0.707	<0.001
Tachygastria ratio (post/pre)	0.638 ± 0.507	1.907 ± 1.784	1.946 ± 1.705	1.159 ± 0.691	<0.001
PRI < 1, n (%)	1 (1.3%)	36 (80%)	60 (100%)	50 (68%)	<0.001
Bradygastria ratio > 1, n (%)	16 (21%)	30 (67%)	44 (73%)	52 (71%)	<0.001
Tachygastria ratio > 1, n (%)	10 (13%)	30 (67%)	39 (65%)	35 (48%)	<0.001

PRI, postprandial response index. A PRI<1 is a descriptive no-increase/no-change reference in this study and not a validated diagnostic threshold. The listed N%, B%, and T% categories do not necessarily sum to 100% because residual or unclassified spectral activity is not displayed; for each group and time point, the displayed categories sum to ≤100%. Ratio variables are means of individual postprandial/preprandial ratios rather than ratios of group-level means.

### 3.3 PRI distribution and postprandial attenuation pattern

The mild-to-moderate anxiety–depression without the FD group offered a distinct contrast. Their preprandial and postprandial normogastria percentages were not frankly abnormal, yet the direction of the meal response was reversed: normogastria decreased after the meal. Mean PRI in this group was lower than in controls, and all participants in this group had PRI < 1. Together, these findings indicate a postprandial attenuation pattern in the mild-to-moderate anxiety–depression without the FD group.

In the healthy control group, PRI was centered above 1, with only one participant below 1. The ratio of 1 was used as a descriptive no-change reference, and the PRI distribution in each group is summarized in Supplementary Table S1.

### 3.4 Heart rate variability profiles

HRV differed across the four groups (Table 3). The controls had the highest SDNN, RMSSD, HF, and LF values and the lowest LF/HF spectral ratio. The FD-only and mild-to-moderate anxiety–depression without FD groups showed lower overall HRV and lower vagal-related indices. The severe anxiety–depression with the FD group had the lowest SDNN, RMSSD, HF, and LF values, together with an elevated LF/HF spectral ratio. Autonomic interpretation focused on RMSSD and HF as vagal-related indices; LF/HF was not interpreted as a direct measure of sympathovagal balance.

**Table 3:** Heart rate variability parameters across groups.

Variable	Healthy controls *N* = 75	FD-only *N* = 45	Mild-to-moderate AnxDep without FD *N* = 60	Severe AnxDep with FD *N* = 73	*p* value
SDNN, ms	141.91 ± 14.24	92.73 ± 6.28	86.15 ± 5.18	64.51 ± 6.41	<0.001
RMSSD, ms	39.99 ± 5.80	16.87 ± 2.32	23.42 ± 2.69	12.64 ± 1.82	<0.001
HF, ms^2^	368.37 ± 97.48	118.65 ± 9.87	134.22 ± 6.65	72.92 ± 9.11	<0.001
LF, ms^2^	497.84 ± 144.02	294.24 ± 16.57	291.21 ± 14.53	183.76 ± 10.41	<0.001
LF/HF spectral ratio	1.35 ± 0.14	2.51 ± 0.34	2.18 ± 0.20	2.55 ± 0.25	<0.001

Values are mean ± SD. SDNN, standard deviation of NN intervals; RMSSD, root mean square of successive differences; HF, high-frequency power; LF, low-frequency power; LF/HF, low-frequency/high-frequency spectral ratio; AnxDep, anxiety–depression.

### 3.5 Sleep quality, medication exposure, and quality-control summaries

Sleep quality showed a clinical gradient. The controls had the lowest PSQI scores, FD-only patients showed mild-to-moderate sleep impairment, and both anxiety–depression groups had poorer sleep. As sleep medication and psychotropic medication exposure were more frequent in anxiety–depression groups, these variables were summarized and used in sensitivity analyses rather than treated as negligible background factors.

EGG quality-control summaries showed that valid preprandial and postprandial recording durations were close to the planned 30-min windows after artifact rejection. No group-level interpretation was based on excluded or incomplete EGG recordings.

### 3.6 Correlation structure among psychological, sleep, HRV, and EGG variables

**Figure 2:**
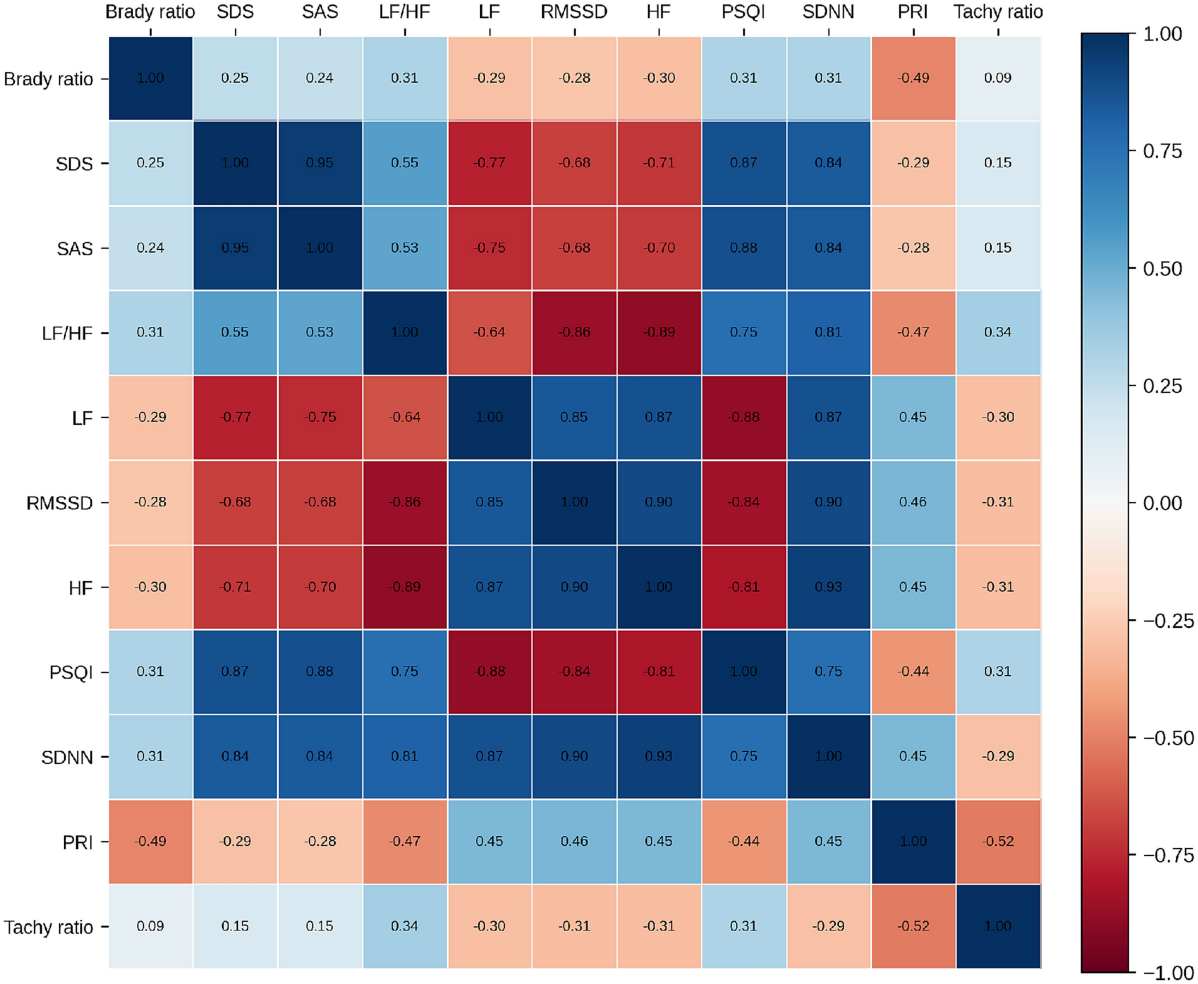
Spearman correlation heatmap of psychological symptoms, sleep quality, heart rate variability, and EGG-derived meal-response variables. The heatmap shows pairwise Spearman correlations among psychological symptom scores, sleep quality, HRV indices, and EGG-derived ratio variables. Positive correlations are shown in blue and negative correlations in red, with darker colors indicating stronger correlations. Values within cells represent Spearman correlation coefficients. SAS, Self-rating Anxiety Scale; SDS, Self-rating Depression Scale; PSQI, Pittsburgh Sleep Quality Index; HRV, heart rate variability; RMSSD, root mean square of successive differences; SDNN, standard deviation of normal-to-normal intervals; LF, low-frequency power; HF, high-frequency power; LF/HF, low-frequency/high-frequency spectral ratio; EGG, electrogastrography; PRI, postprandial response index.

Spearman correlations showed a coherent association pattern (Figure 2). SAS, SDS, and PSQI were inversely correlated with RMSSD and HF and positively correlated with the LF/HF spectral ratio. Psychological and sleep measures were also inversely correlated with PRI. RMSSD was positively correlated with PRI, whereas dysrhythmic ratios generally showed the opposite direction. Overall, greater psychological and sleep symptom load was associated with lower vagal-related HRV and weaker postprandial slow-wave response.

### 3.7 Regression models, collinearity diagnostics, and sensitivity analyses

Collinearity diagnostics showed high VIF values for SAS, SDS, and PSQI when they were included simultaneously in the same model. Therefore, the main analyses used separate parsimonious models adjusted for age, sex, and BMI. Coefficient instability was most evident for SAS: in the exploratory simultaneous model, the SAS coefficients were +0.278 for RMSSD, +0.004 for PRI, and −0.281 for FD symptom score, whereas the corresponding separate-model coefficients were −0.400, −0.002, and +0.402, respectively. SDS and PSQI retained their coefficient directions across the two specifications. Given the high VIFs for SAS and SDS, this pattern was interpreted as reflecting suppression/coefficient instability.

Sensitivity analyses showed that the AnxDep-noFD vs. control differences in PRI and RMSSD remained after adjustment for BMI or weight, after medication adjustment, after excluding SSRI/SNRI users or sleep-medication users, and in analyses restricted to volunteer/outpatient participants. For RMSSD, the group+age+sex+BMI model had R^2^ = 0.902 and adjusted R^2^ = 0.900. After adding any psychotropic medication and sleep medication use, R^2^ remained at 0.902 to the reported precision, and adjusted R^2^ was 0.899. The three RMSSD group coefficients changed by less than 2% after medication adjustment. Full model specifications and adjusted R^2^ values are shown in Supplementary Table S8.

## 4 Discussion

This study linked anxiety–depression symptoms and poor sleep with lower vagal-related HRV and an altered postprandial EGG response. The most notable finding was observed in participants with mild-to-moderate anxiety–depression but no FD symptoms: absolute normogastria values were not frankly abnormal, yet normogastria decreased after meal stimulation, producing a PRI < 1 in every participant in this group (Table 4).

**Table 4:** Separate regression models for PRI, RMSSD, and FD symptom score.

Outcome	Model	Predictor	Beta	95% CI	*p* value	Adjusted R^2^	n
PRI	SAS + age + sex + BMI	SAS	−0.002	−0.003 to −0.001	0.005	0.020	253
PRI	SDS + age + sex + BMI	SDS	−0.002	−0.003 to −0.001	0.004	0.023	253
PRI	PSQI + age + sex + BMI	PSQI	−0.012	−0.016 to −0.007	<0.001	0.093	253
RMSSD	SAS + age + sex + BMI	SAS	−0.400	−0.460 to −0.340	<0.001	0.419	253
RMSSD	SDS + age + sex + BMI	SDS	−0.408	−0.465 to −0.351	<0.001	0.452	253
RMSSD	PSQI + age + sex + BMI	PSQI	−1.726	−1.911 to −1.541	<0.001	0.583	253
FD symptom score (FD subgroup)	SAS + age + sex + BMI	SAS	0.402	0.326 to 0.478	<0.001	0.563	118
FD symptom score (FD subgroup)	SDS + age + sex + BMI	SDS	0.398	0.323 to 0.473	<0.001	0.563	118
FD symptom score (FD subgroup)	PSQI + age + sex + BMI	PSQI	1.591	1.208 to 1.974	<0.001	0.462	118

Models were adjusted for age, sex, and BMI. Separate models were used to reduce interpretive problems caused by high collinearity among SAS, SDS, and PSQI. The FD symptom score models were restricted to participants with FD (*n* = 118).

PRI captures the shift from fasting to postprandial gastric myoelectrical activity. In controls, the meal increased the proportion of normal slow waves and reduced dysrhythmic activity, consistent with a coordinated adaptive response. A low PRI indicates that this transition was blunted or reversed within the measurement framework. Because PRI is a ratio that depends on both fasting and postprandial normogastria, it should be interpreted as an exploratory meal-response index rather than a diagnostic threshold or disease-stage marker.

The HRV results provide an autonomic context for the EGG findings. RMSSD and HF were lower across the FD and anxiety–depression phenotypes, with the largest reduction in the severe anxiety–depression with the FD group. Because RMSSD and HF are related to cardiac vagal modulation, this profile is consistent with reduced vagal-related autonomic flexibility. LF/HF was retained only as a relative spectral ratio and was not used to infer sympathovagal balance.

### 4.1 Potential mechanisms

Several mechanisms may plausibly link anxiety–depression, poor sleep, vagal-related HRV, and postprandial gastric slow-wave attenuation. Psychological distress and sleep disturbance may increase central arousal and stress-system activation, reduce vagal-related regulation, alter pain thresholds, and destabilize interoceptive processing. Vagal pathways regulate gastric accommodation, antral coordination, secretion, inflammatory tone, and visceral afferent signaling, and could plausibly influence meal-related rhythm adaptation. Surface EGG, however, records gastric rhythm rather than gastric emptying, accommodation, or contractile force, so this mechanistic interpretation remains associative and requires confirmation with longitudinal or interventional designs.

Sleep quality was closely related to both RMSSD and PRI, supporting the relevance of sleep disturbance to autonomic and meal-related gastric rhythm measures. Poor sleep may increase arousal, reduce vagal modulation, alter pain processing, and destabilize interoceptive regulation. Medication exposure was more frequent in the anxiety–depression groups and may affect HRV, sleep architecture, and gastrointestinal physiology. Because medication exposure was highly aligned with clinical phenotype and recruitment setting, the medication-adjusted models should be interpreted as sensitivity analyses rather than definitive pharmacologic adjustment. The minimal change in RMSSD group coefficients suggests that broad medication indicators added little unique variance beyond group and baseline covariates, but residual confounding by indication, drug class, dose, duration, adherence, and timing remains possible.

The regression strategy was intended to reduce overinterpretation of highly collinear psychological and sleep predictors. SAS, SDS, and PSQI were strongly interrelated; when included simultaneously, their coefficients can change direction because of suppression and unstable variance partitioning. The SAS sign reversal occurred for all three outcomes: simultaneous-model coefficients of +0.278 for RMSSD, +0.004 for PRI, and −0.281 for FD symptom score changed to −0.400, −0.002, and +0.402, respectively, in the corresponding separate models, while SDS and PSQI did not reverse. This asymmetric pattern supports the suppression/coefficient-instability interpretation. Separate models provide a clearer descriptive summary, but they should not be interpreted as independent causal-effect models.

The current observation is best viewed as a hypothesis-generating finding. Meal-related gastric myoelectrical adaptation may be altered in some individuals with anxiety–depression symptoms who do not meet FD criteria at enrollment. This supports future longitudinal research in patients with persistent psychological and sleep symptoms, particularly when meal-related discomfort is emerging but remains subthreshold.

### 4.2 Limitations

Several limitations should be noted. First, the study was cross-sectional and could not determine temporal order. A PRI < 1 may reflect behavioral, dietary, autonomic, medication-related, nutritional, or selection factors, and its prospective significance requires longitudinal validation. Second, the group structure was not fully factorial, so anxiety–depression severity, FD status, and their interaction cannot be separated. Third, recruitment source differed across groups, and severe anxiety–depression with FD participants were more likely to be recruited from inpatient settings, which may introduce selection bias. Prior psychiatric history and recent stressful events were recorded descriptively and were not measured using standardized life-event instruments.

Fourth, medication exposure was summarized at the class level and included in sensitivity analyses, but residual confounding by dose, duration, exact timing, adherence, and confounding by indication cannot be excluded. Fifth, weight and BMI differed across groups. Although BMI and weight adjustment were performed, additional nutritional and weight-loss variables were descriptive and should not be interpreted as a complete nutritional assessment. Sixth, surface EGG is sensitive to motion artifacts, electrode placement, signal processing, and analytical choices; 30-min recording windows remain relatively short, and EGG cannot replace direct motility testing or high-resolution mapping. Because the EGG quality-control process was consensus-based and was not designed as a prespecified independent reliability study, formal inter-reviewer reliability could not be estimated. Seventh, PRI = 1 was used as a descriptive no-change reference; its reproducibility, diagnostic accuracy, and clinically meaningful change require independent validation. Eighth, no *a priori* sample-size calculation was performed. The achieved-sample sensitivity analysis indicates that the study was powered only for the stated small-to-moderate or moderate effect ranges, whereas very small effects, medication-class effects, and sparse subgroup contrasts may have been missed.

Future longitudinal studies should assess patients with anxiety–depression but no FD using standardized EGG, HRV, sleep measures, detailed medication data, nutritional measures, and repeated symptom follow-up. Such studies could determine whether a PRI < 1 is associated with incident FD, whether improvement in sleep or vagal-related HRV is accompanied by normalization of PRI, and whether PRI adds information beyond SAS, SDS, PSQI, medication exposure, and demographic variables.

## 5 Conclusion

Anxiety–depression symptoms were associated with lower vagal-related HRV, poorer sleep quality, and altered electrogastrographic adaptation to meal stimulation. The most distinctive finding was a PRI < 1 in all participants with mild-to-moderate anxiety–depression but without FD symptoms, despite preserved absolute normogastria values. This postprandial attenuation pattern should be interpreted as an exploratory cross-sectional finding. PRI and HRV may serve as research endpoints for future longitudinal studies of sleep and autonomic regulation in FD-related gut–brain interaction.

## Data Availability

The datasets presented in this study can be found in online repositories. The names of the repository/repositories and accession number(s) can be found in the article/Supplementary material.
