# Supplementary material for: Postprandial attenuation of gastric slow waves in anxiety–depression, with and without functional dyspepsia: associations with heart rate variability and sleep quality
Source: Front Med (Lausanne). 2026 Jul 27;13:1890716. doi: 10.3389/fmed.2026.1890716 (PMC13453828; doi:10.3389/fmed.2026.1890716)
Supplement: Supplementary file 1 [file Table_1.docx]

**Postprandial attenuation of gastric slow waves in anxiety-depression with and without functional dyspepsia: links with heart rate variability and sleep quality**

Lin Du1,2, Jie Yang1,*, Li Jiang1,3, Guiying Zeng1,4, Yanping Shu5, Bin Bi5

1 Department of Gastroenterology, Affiliated Hospital of Guizhou Medical University, Guiyang 550004, Guizhou, China

2 Department of Gastroenterology, Anshun People's Hospital, Anshun 561000, Guizhou, China

3 Department of Gastroenterology, The Second People's Hospital of Guizhou Province, Guiyang 550004, Guizhou, China

4 Department of Gastroenterology, Qixingguan District People's Hospital of Bijie City, Bijie 551700, Guizhou, China

5 Department of Clinical Psychology, The Second People's Hospital of Guizhou Province, Guiyang 550004, Guizhou, China

Corresponding Author: Dr. Jie Yang; Email: yangjielaile@126.com

**Supplementary Table S1. PRI reference distribution by group.**

| **Group** | **n** | **mean ± SD** | **median** | **IQR** | **min** | **max** | **2.5th percentile** | **97.5th percentile** | **PRI<1 n (%)** |
| --- | --- | --- | --- | --- | --- | --- | --- | --- | --- |
| Healthy controls | 75 | 1.145 ± 0.086 | 1.151 | 1.091 to 1.202 | 0.960 | 1.340 | 1.003 | 1.314 | 1 (1.3%) |
| FD-only | 45 | 0.851 ± 0.160 | 0.826 | 0.742 to 0.945 | 0.605 | 1.163 | 0.621 | 1.139 | 36 (80.0%) |
| Mild-to-moderate AnxDep without FD | 60 | 0.874 ± 0.066 | 0.881 | 0.831 to 0.921 | 0.720 | 0.985 | 0.734 | 0.982 | 60 (100.0%) |
| Severe AnxDep with FD | 73 | 0.950 ± 0.239 | 0.893 | 0.779 to 1.087 | 0.592 | 1.596 | 0.625 | 1.484 | 50 (68.5%) |

PRI<1 is presented as a descriptive no-change reference only. It is not a validated diagnostic or predictive threshold.

**Supplementary Table S2. Medication exposure by group.**

| **Medication category** | **Healthy controls N=75** | **FD-only N=45** | **Mild-to-moderate AnxDep without FD N=60** | **Severe AnxDep with FD N=73** |
| --- | --- | --- | --- | --- |
| Any psychotropic medication | 1 (1.3%) | 3 (6.7%) | 42 (70.0%) | 68 (93.2%) |
| SSRI | 0 (0%) | 0 (0%) | 12 (20.0%) | 35 (47.9%) |
| SNRI | 0 (0%) | 0 (0%) | 11 (18.3%) | 16 (21.9%) |
| SSRI or SNRI, unique users | 0 (0%) | 0 (0%) | 18 (30.0%) | 45 (61.6%) |
| Benzodiazepine | 0 (0%) | 2 (4.4%) | 21 (35.0%) | 26 (35.6%) |
| Non-benzodiazepine hypnotic | 1 (1.3%) | 1 (2.2%) | 16 (26.7%) | 44 (60.3%) |
| Antipsychotic | 0 (0%) | 0 (0%) | 1 (1.7%) | 22 (30.1%) |
| Mood stabilizer | 0 (0%) | 0 (0%) | 2 (3.3%) | 1 (1.4%) |
| PPI | 1 (1.3%) | 15 (33.3%) | 2 (3.3%) | 23 (31.5%) |
| H2 blocker | 2 (2.7%) | 5 (11.1%) | 2 (3.3%) | 13 (17.8%) |
| Current prokinetic | 0 (0%) | 0 (0%) | 0 (0%) | 0 (0%) |
| Current anticholinergic | 0 (0%) | 0 (0%) | 0 (0%) | 0 (0%) |
| Current tricyclic antidepressant | 0 (0%) | 0 (0%) | 0 (0%) | 0 (0%) |
| Sleep medication use | 1 (1.3%) | 3 (6.7%) | 29 (48.3%) | 56 (76.7%) |

Medication categories were not mutually exclusive. The unique SSRI/SNRI row was used for the exclusion sensitivity analysis. Current prokinetic, anticholinergic and tricyclic antidepressant use was absent after exclusion or washout rules. Medication classes were abstracted from available source records and rechecked during data verification; dose, duration, adherence, and exact timing of last intake were not consistently available.

**Supplementary Table S3. Standardized test meal protocol summary.**

| **Item** | **Healthy controls N=75** | **FD-only N=45** | **Mild-to-moderate AnxDep without FD N=60** | **Severe AnxDep with FD N=73** |
| --- | --- | --- | --- | --- |
| Test meal components | Steamed bread 100 g + boiled egg 50 g + milk 250 mL | Same | Same | Same |
| Estimated total energy | ~430 kcal | ~430 kcal | ~430 kcal | ~430 kcal |
| Approximate carbohydrate/protein/fat | 58 g / 19 g / 14 g | Same | Same | Same |
| Approximate meal amount | ~150 g solid food + 250 mL milk | Same | Same | Same |
| Standardized meal protocol | Same meal prescribed by laboratory protocol | Same | Same | Same |
| Target meal-consumption window | Within 15 min | Same | Same | Same |
| Fasting requirement | Overnight fast >=8 h | Same | Same | Same |
| Pre-test restrictions | Avoid caffeine/alcohol within 24 h, smoking on testing morning, and strenuous activity within 24 h | Same | Same | Same |
| Adherence reporting | Protocol requirements checked verbally before EGG testing; not analyzed as quantitative adherence outcomes | Same | Same | Same |

Estimated energy is approximate; 58 g carbohydrate, 19 g protein and 14 g fat correspond to approximately 434 kcal using 4/4/9 kcal per gram conversion. Fasting, restriction, and meal-consumption entries are laboratory protocol requirements and were not used as quantitative adherence outcomes.

**Supplementary Table S4. EGG signal-processing and quality-control summary.**

| **Item** | **Healthy controls N=75** | **FD-only N=45** | **Mild-to-moderate AnxDep without FD N=60** | **Severe AnxDep with FD N=73** |
| --- | --- | --- | --- | --- |
| EGG device | Meda electrogastrography system | Same | Same | Same |
| Electrode placement | Standard epigastric three-electrode placement | Same | Same | Same |
| Sampling rate | 1 Hz | 1 Hz | 1 Hz | 1 Hz |
| Analysis/filter band | 0.5-9.0 cpm | Same | Same | Same |
| Bradygastria/normogastria/tachygastria bands | 1.0-2.0 / 2.0-4.0 / 4.0-9.0 cpm | Same | Same | Same |
| Planned pre-recording, min | 30 | 30 | 30 | 30 |
| Planned post-recording, min | 30 | 30 | 30 | 30 |
| Valid pre-recording after artifact rejection, min | 28.87 ± 0.43 | 28.75 ± 0.36 | 28.70 ± 0.36 | 28.25 ± 0.38 |
| Valid post-recording after artifact rejection, min | 28.81 ± 0.44 | 28.45 ± 0.49 | 28.38 ± 0.49 | 27.96 ± 0.58 |
| Artifact percentage before meal | 3.77 ± 1.44 | 4.17 ± 1.20 | 4.33 ± 1.22 | 5.83 ± 1.28 |
| Artifact percentage after meal | 3.97 ± 1.46 | 5.17 ± 1.63 | 5.40 ± 1.62 | 6.80 ± 1.92 |
| Quality-control approach | Primary artifact review with second-review consensus for borderline/artifact-prone recordings | Same | Same | Same |
| Excluded from EGG analysis | 0 (0%) | 0 (0%) | 0 (0%) | 0 (0%) |

Artifact percentage was calculated as (planned duration minus valid duration) divided by planned duration. Recording acceptability and artifact rejection were finalized by consensus before spectral summaries were analyzed; the consensus QC process was not designed as a prespecified independent reliability study.

**Supplementary Table S5. Descriptive recruitment source, weight/nutritional variables and recent stress history.**

| **Variable** | **Healthy controls N=75** | **FD-only N=45** | **Mild-to-moderate AnxDep without FD N=60** | **Severe AnxDep with FD N=73** |
| --- | --- | --- | --- | --- |
| Recruitment source: volunteer/outpatient/inpatient | 75 / 0 / 0 | 0 / 37 / 8 | 0 / 37 / 23 | 0 / 24 / 49 |
| Recent weight loss, n (%) | 2 (2.7%) | 9 (20.0%) | 13 (21.7%) | 26 (35.6%) |
| Unintentional weight loss, n (%) | 1 (1.3%) | 6 (13.3%) | 6 (10.0%) | 15 (20.5%) |
| Weight loss, kg | 0.07 ± 0.46 | 0.40 ± 0.98 | 0.51 ± 1.11 | 0.69 ± 1.33 |
| Albumin, g/L | 43.91 ± 2.19 | 42.87 ± 1.88 | 43.19 ± 2.82 | 41.18 ± 2.47 |
| Hemoglobin, g/L | 129.89 ± 11.48 | 130.30 ± 12.13 | 130.15 ± 11.88 | 130.10 ± 10.82 |
| FD-scale appetite-loss item | 0.00 ± 0.00 | 1.51 ± 0.83 | 0.00 ± 0.00 | 2.01 ± 0.79 |
| Prior psychiatric history, n (%) | 0 (0%) | 0 (0%) | 24 (40.0%) | 40 (54.8%) |
| Recent stressful event within 6 months, n (%) | 8 (10.7%) | 4 (8.9%) | 27 (45.0%) | 42 (57.5%) |

The appetite-loss item refers to the structured FD symptom scale rather than a general mood-related appetite scale. Weight/nutritional variables are descriptive summaries used to contextualize group differences rather than definitive nutritional trajectories. Prior psychiatric history and recent stressful events were recorded descriptively during clinical interview and were not standardized by a formal life-event scale.

**Supplementary Table S6. Sample-size and design considerations.**

| **Item** | **Value/description** |
| --- | --- |
| Study design | Exploratory single-center cross-sectional observational phenotype comparison |
| Enrollment/sample-size basis | All eligible participants during the prespecified recruitment period; no formal a priori calculation |
| Sensitivity software/settings | G*Power 3.1.9.7; alpha=0.05; power=0.80; pairwise test two-sided |
| Final sample size | 253 participants: 75 HC, 45 FD-only, 60 AnxDep-noFD, 73 severe AnxDep+FD |
| Four-group omnibus sensitivity | N=253, four groups; minimum detectable Cohen f=0.209 |
| Key HC vs AnxDep-noFD contrast | n=75 vs n=60; minimum detectable Cohen d=0.489 at alpha=0.05 and d=0.611 at Bonferroni alpha=0.0083 |
| Full-sample regression sensitivity | Fixed-model R² increase; one tested predictor, four total predictors; N=253; minimum detectable f²=0.031 |
| FD-subgroup regression sensitivity | Fixed-model R² increase; one tested predictor, four total predictors; n=118; minimum detectable f²=0.068 |
| FD-model predictor density | 118/4 = 29.5 observations per predictor |
| Interpretation | Sensitive to small-to-moderate omnibus/regression effects and moderate pairwise effects; limited for very small or sparse effects. |
| Main inferential caution | Exploratory sensitivity assessment; it does not establish causal or predictive validity and does not replace an a priori calculation |

The sensitivity analysis used alpha=0.05 and 80% power; the pairwise threshold was two-sided. Effect sizes were not estimated from the observed study results. The four-group calculation used a fixed-effects omnibus test; regression calculations used the fixed-model R²-increase framework with one tested predictor and four total predictors.

**Supplementary Table S7. Collinearity diagnostics and coefficient-instability summary for the full model predictors.**

| **Model** | **Predictor** | **R² against other predictors** | **VIF** | **Tolerance** | **Interpretation** |
| --- | --- | --- | --- | --- | --- |
| Full model predictors | SAS_total | 0.964 | 27.98 | 0.036 | High collinearity; separate models preferred |
| Full model predictors | SDS_total | 0.965 | 28.62 | 0.035 | High collinearity; separate models preferred |
| Full model predictors | PSQI_total | 0.843 | 6.38 | 0.157 | High collinearity; separate models preferred |
| Full model predictors | age | 0.018 | 1.02 | 0.982 | Low collinearity |
| Full model predictors | sex_bin | 0.015 | 1.02 | 0.985 | Low collinearity |
| Full model predictors | BMI | 0.139 | 1.16 | 0.861 | Low collinearity |

Values below are simultaneous-model versus separate-model coefficients. The SAS coefficient reversed sign for RMSSD (+0.278 vs -0.400), PRI (+0.004 vs -0.002), and FD symptom score (-0.281 vs +0.402), whereas SDS and PSQI retained their directions. Given the very high SAS/SDS VIFs, these reversals were treated as suppression/coefficient instability and were not interpreted as independent effects.

**Supplementary Table S8. Sensitivity analyses with explicit model specifications for body weight, medication exposure, and recruitment source.**

| **Model / analysis set** | **Outcome** | **Contrast** | **Estimate** | **95% CI** | **P value** | **Adjusted R²** | **n** |
| --- | --- | --- | --- | --- | --- | --- | --- |
| Model A | PRI | FD-only vs HC | -0.294 | -0.352 to -0.236 | <0.001 | 0.402 | 253 |
| Model A | PRI | AnxDep-noFD vs HC | -0.270 | -0.322 to -0.219 | <0.001 | 0.402 | 253 |
| Model A | PRI | Severe AnxDep+FD vs HC | -0.195 | -0.247 to -0.143 | <0.001 | 0.402 | 253 |
| Model B | PRI | FD-only vs HC | -0.295 | -0.353 to -0.237 | <0.001 | 0.404 | 253 |
| Model B | PRI | AnxDep-noFD vs HC | -0.272 | -0.324 to -0.220 | <0.001 | 0.404 | 253 |
| Model B | PRI | Severe AnxDep+FD vs HC | -0.197 | -0.249 to -0.145 | <0.001 | 0.404 | 253 |
| Model C | PRI | FD-only vs HC | -0.295 | -0.354 to -0.236 | <0.001 | 0.398 | 253 |
| Model C | PRI | AnxDep-noFD vs HC | -0.282 | -0.350 to -0.214 | <0.001 | 0.398 | 253 |
| Model C | PRI | Severe AnxDep+FD vs HC | -0.209 | -0.288 to -0.130 | <0.001 | 0.398 | 253 |
| Model A | RMSSD | FD-only vs HC | -23.196 | -24.583 to -21.808 | <0.001 | 0.900 | 253 |
| Model A | RMSSD | AnxDep-noFD vs HC | -16.586 | -17.854 to -15.318 | <0.001 | 0.900 | 253 |
| Model A | RMSSD | Severe AnxDep+FD vs HC | -27.343 | -28.598 to -26.089 | <0.001 | 0.900 | 253 |
| Model C | RMSSD | FD-only vs HC | -23.219 | -24.614 to -21.825 | <0.001 | 0.899 | 253 |
| Model C | RMSSD | AnxDep-noFD vs HC | -16.850 | -18.531 to -15.169 | <0.001 | 0.899 | 253 |
| Model C | RMSSD | Severe AnxDep+FD vs HC | -27.738 | -29.673 to -25.804 | <0.001 | 0.899 | 253 |
| Model D: no SSRI/SNRI users | PRI | AnxDep-noFD vs HC | -0.271 | -0.323 to -0.219 | <0.001 | 0.515 | 190 |
| Model D: no SSRI/SNRI users | RMSSD | AnxDep-noFD vs HC | -16.774 | -18.376 to -15.173 | <0.001 | 0.886 | 190 |
| Model E: no sleep-medication users | PRI | AnxDep-noFD vs HC | -0.292 | -0.349 to -0.235 | <0.001 | 0.536 | 164 |
| Model E: no sleep-medication users | RMSSD | AnxDep-noFD vs HC | -16.659 | -18.487 to -14.830 | <0.001 | 0.874 | 164 |
| Model F: volunteer/outpatient only | PRI | AnxDep-noFD vs HC | -0.270 | -0.317 to -0.223 | <0.001 | 0.589 | 173 |
| Model F: volunteer/outpatient only | RMSSD | AnxDep-noFD vs HC | -16.550 | -18.258 to -14.843 | <0.001 | 0.876 | 173 |

HC = healthy controls; FD = functional dyspepsia; AnxDep = anxiety-depression; RMSSD = root mean square of successive differences; PRI = postprandial response index. Each row reports an ordinary least-squares model with continuous PRI or RMSSD. Group was represented by three 0/1 indicators with HC as the reference; the estimate is the unstandardized coefficient for the named group indicator. Age was entered in years, BMI in kg/m², and weight in kg; sex and medication-use variables were entered as binary indicators. Model A: all participants, outcome ~ group + age + sex + BMI. Model B: all participants, outcome ~ group + age + sex + weight (BMI omitted). Model C: all participants, outcome ~ group + age + sex + BMI + any psychotropic medication + sleep-medication use. Model D: participants not using SSRI/SNRI, outcome ~ group + age + sex. Model E: participants not using sleep medication, outcome ~ group + age + sex. Model F: volunteer/outpatient participants only, outcome ~ group + age + sex. Adjusted R² is for the full model. For RMSSD, Model A had R²=0.902 (adjusted R²=0.900) and Model C had R²=0.902 at the reported precision (adjusted R²=0.899). Because medication exposure was strongly aligned with group, Model C estimates medication effects only from variance not already represented by group and the other covariates.
